# Effects of Infection-Induced Migration Delays on the Epidemiology of Avian Influenza in Wild Mallard Populations

**DOI:** 10.1371/journal.pone.0026118

**Published:** 2011-10-18

**Authors:** Stephen J. Galsworthy, Quirine A. ten Bosch, Bethany J. Hoye, Johan A. P. Heesterbeek, Marcel Klaassen, Don Klinkenberg

**Affiliations:** 1 Faculty of Veterinary Medicine, Utrecht University, Utrecht, The Netherlands; 2 Department of Biological Sciences, University of Notre Dame, Notre Dame, Indiana, United States of America; 3 Department of Animal Ecology, Netherlands Institute of Ecology (NIOO-KNAW), Wageningen, The Netherlands; 4 Centre for Integrative Ecology, Deakin University, Geelong, Victoria, Australia; University of Georgia, United States of America

## Abstract

Wild waterfowl populations form a natural reservoir of Avian Influenza (AI) virus, and fears exist that these birds may contribute to an AI pandemic by spreading the virus along their migratory flyways. Observational studies suggest that individuals infected with AI virus may delay departure from migratory staging sites. Here, we explore the epidemiological dynamics of avian influenza virus in a migrating mallard (*Anas platyrhynchos*) population with a specific view to understanding the role of infection-induced migration delays on the spread of virus strains of differing transmissibility. We develop a host-pathogen model that combines the transmission dynamics of influenza with the migration, reproduction and mortality of the host bird species. Our modeling predicts that delayed migration of individuals influences both the timing and size of outbreaks of AI virus. We find that (1) delayed migration leads to a lower total number of cases of infection each year than in the absence of migration delay, (2) when the transmission rate of a strain is high, the outbreak starts at the staging sites at which birds arrive in the early part of the fall migration, (3) when the transmission rate is low, infection predominantly occurs later in the season, which is further delayed when there is a migration delay. As such, the rise of more virulent AI strains in waterfowl could lead to a higher prevalence of infection later in the year, which could change the exposure risk for farmed poultry. A sensitivity analysis shows the importance of generation time and loss of immunity for the effect of migration delays. Thus, we demonstrate, in contrast to many current transmission risk models solely using empirical information on bird movements to assess the potential for transmission, that a consideration of infection-induced delays is critical to understanding the dynamics of AI infection along the entire flyway.

## Introduction

Waterfowl, and notably dabbling ducks (genus *Anas*), are considered to form a natural reservoir of influenza A viruses [1]. Of the possible combinations of the 16 HA and 9 NA antigenic subtypes of influenza, nearly all have been found in wild dabbling ducks [2]–[5]. Strains causing disease in humans, poultry and other animals, including the H5 and H7 highly pathogenic avian influenza (HPAI) strains, have their low pathogenic precursors in wild birds [6], [7]. Generally, most cases of influenza in waterfowl are low-pathogenic (LPAI) strains. In North America, they are predominantly observed just after breeding, during fall migration, with prevalences dropping in December when the birds are at the wintering grounds [2]. This could differ however between strains of varying pathogenicity or transmissibility. The interplay between infectious disease dynamics and animal migration is not very well understood [8]. Fundamental understanding of the origin and spread of influenza viruses through wild bird populations is essential for designing strategies to recognise threats early and to minimise the risk of outbreaks.

In order for migratory birds to spread avian influenza over large geographic regions, the infection must not affect their behavior or physiology in ways that compromise their ability to undertake sustained flight [9]. However, the effect of influenza virus upon the physiological characteristics and migration behaviour of wild birds is unclear. Laboratory based studies [10]–[12] have shown that for some, but not all HPAI strains, infection is subclinical in some species of waterfowl. However there is no evidence to show whether HPAI infection is asymptomatic in free-living birds of these species. In wild migratory Bewick's swans, Van Gils et al. [13] found that infection with LPAI may lead to delayed departure from wintering sites, shorter distances travelled and fuelling and feeding at reduced rates. Latorre-Margalef et al. [14] found that LPAI did reduce the body mass of mallards but they could not find a general effect of infection on staging time. Presently, no such data exist on the effect of HPAI on wild ducks [9].

Mallards (*Anas platyrhynchos*) are the most abundant species of dabbling duck over much of Eurasia and North America [15]. In Europe, wild mallards often migrate over long distances between their northern breeding sites and their southern wintering sites. Fears exist that mallards may contribute to an AI-pandemic by spreading the virus along these migratory pathways [3], [16], [17]. These seasonal migrations are not, however, achieved in a single long distance flight. Instead both spring (northward) and autumn (southward) migration are characterised by a number of flight periods interspersed by periods of refuelling. During these refuelling periods birds congregate in large numbers at staging sites [18]. The clinical effects of infection may lead to a delay in departure from these staging sites for infected individuals. One hypothesis is that delayed migration might facilitate virus transmission because infected individuals remain longer on crowded staging sites where they are in close proximity to susceptible ducks [14]. However the contrary may also be true whereby infected individuals become isolated from the main susceptible population as a result of delay [8]. Considering the potential role of mallards in the spread of AI, it is vital to understand infection dynamics in mallards, and how these may change if a new strain affects transmissibility or clinical outcome of infection. Mathematical modelling provides a means to investigate this.

In this paper we use population modelling to study how AI strains that induce varying delays in migration (as a proxy for a suite of possible pathogenic effects) and different transmissibility between birds could spread in a migrating mallard population. Our new host-pathogen model combines the transmission dynamics of influenza with the migration, reproduction and mortality of the host bird species. We show where and when the highest number of infected birds is to be expected and how this is affected by the rate of virus transmission and the migration delay due to infection.

## Methods

Our model describes the spread of avian influenza in a typical population (around 5,000 individuals) of mallard ducks (*Anas platyrhynchos*) that migrates twice per year between a northern breeding ground (Northern Scandinavia) and a southern wintering ground (The Netherlands). During these migration periods, the birds rest at a handful of staging sites in order to feed and recover. A satellite telemetry study by Yamaguchi et al. [18] of mallards which spend winter in Japan suggests that the mean number of staging sites is between 1.3 and 3 depending on the chosen location for breeding and that mallards stay for one to four weeks at each staging site between short travel periods of a few days.

### Population dynamics

**Figure 1:**
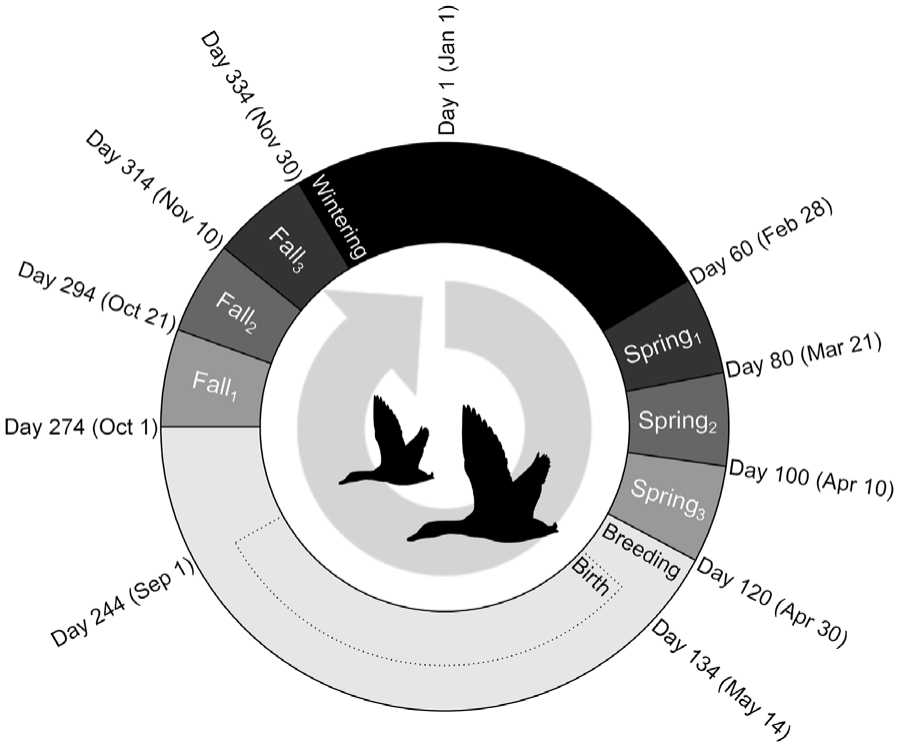
The annual migration cycle of mallards between eight distinct patches. The size of each sector indicates the relative amount of time that birds spend in each of the eight patches. The dates around the outside describe the date from which birds are able to leave one patch and migrate to the next. In each calendar year the birds start in the wintering patch (which we take to be patch 1). The migration rate is defined such that between the fixed dates of arrival to and departure from a particular patch *i*, the migration rate *m_i_* = 0, whilst at all other times *m_i_* = 1. Birth takes place at the breeding grounds (patch 5) from two weeks after arrival and ceases one month before departure and is shown by the dotted sector.

We consider a situation where the birds pause during each biannual migration at three distinct sites leading to a model with eight distinct patches (one for wintering, three during spring migration, one for breeding, and three during fall sequentially referenced as patches *i* = 1 to *i* = 8), see Figure 1. Having arrived at a particular patch, the birds remain there until the date arises in which they may move on, as shown in Figure 1. In the model, this means that the migration rate *m_i_* (leaving patch *i*) is equal to 0 in the time interval that the birds are supposed to be there, and equal to *m* = 1 outside the interval. The transition occurs by a step function. After the interval, birds which are “healthy enough” to migrate are free to move to the next patch. Birds which are not able to migrate stay at their current location until they recover sufficiently to be able to migrate. We consider the birds to stay at each migration staging site for a minimum of 20 days, to spend at least 3 months at the wintering patch, and to spend the rest of their time at the breeding grounds. Migration is modelled so that the average migration time between neighbouring patches is one day.

In the model, ducks experience natural mortality, at rate *m*, at all patches and additional mortality due to hunting, at rate *m_h_*, at the fall migration and wintering grounds. Mortality rates have been approximated using the estimated average life expectancy of mallards of 2.27 years [19] and a 30% contribution of hunting to the total mortality [20].

Birth occurs from six weeks after arrival on the breeding grounds and ceases one month before departure to allow time for ducklings to grow sufficiently for migration (Figure 1). During these 54 days, 40 new birds enter the population each day (*b* = 40), which sums to 2160 new birds per year, about one per adult female. These are only the ducklings that survive to adulthood. Due to this birth rate, the population size fluctuates around 4000–6000 individuals, the size chosen for our typical mallard population.

### Dynamics of infection

**Figure 2:**
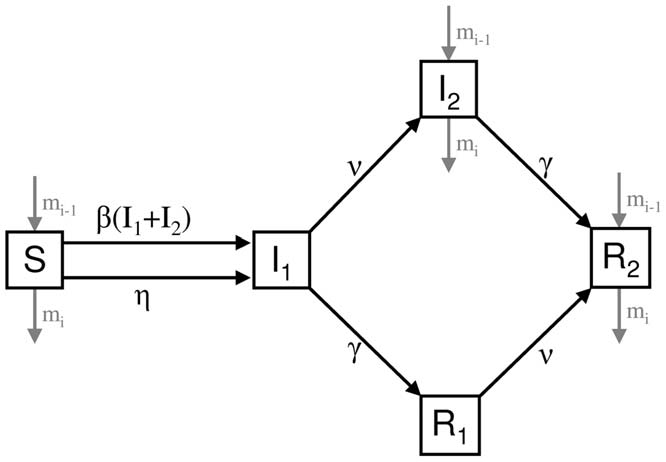
Flowchart showing the movement of individuals between compartments within each patch as described by the model (1). Birds belong to one of five compartments: Susceptible (*S*), infected and unable to migrate (*I*_1_), infected and able to migrate (*I*_2_), recovered and unable to migrate (*R*_1_), or recovered and able to migrate (*R*_2_). Susceptible birds become infected via either direct or environmental transmission, with rates determined by *b* and *h* respectively (Table 1). Birds regain the ability to migrate at rate *n*, and recover from infection at rate *g*. Migration is only possible for birds in classes *S*, *I*_2_ and *R*_2_ such that in each of these three compartments birds enter at rate *m_i_*_−1_ and leave at rate *m_i_*. Natural mortality, at rate *m*, occurs equally across all five compartments, yet is not shown in this diagram for clarity. Mortality due to hunting, occurring at rate *m_h_* in the winter and fall patches, is also not shown here.

Within each patch the infection transmission is modelled using an *SIR*-type model. We take the standard compartments: susceptibles (*S*), infected (*I*) and recovered (*R*) but divide both the infected and recovered classes into two subclasses as shown in Figure 2. *I*_1_ and *R*_1_ contain birds which are respectively infected or recovered but cannot migrate, whereas *I*_2_ and *R*_2_ contain infected or recovered birds which are able to migrate. We did not include multiple strains and cross-protection, which would make the model too complex for our aim. Instead, in the baseline model we assumed no loss of immunity. As another extreme, in a sensitivity analysis, we considered an alternative model in which all birds lost their immunity at the end of each breeding season.

Influenza virus transmission occurs mainly through the environment: infectious birds shed virus, which becomes available for infection of susceptible birds. Because most excreted virus is short-lived in the environment [21], transmission is modelled with a direct-contact transmission term. Apart from this direct transmission, we included a low background transmission rate due to long-term virus survival or contact with other populations (see below). Direct transmission from bird to bird within the population is assumed to be density dependent within the range of population sizes simulated (4000–6000 birds). This means that an infectious bird is likely to infect more birds if the number of susceptible birds increases rather than the proportion. As mallards exhibit more solitary behaviour whilst breeding, contact rates at the breeding grounds (patch 5) are assumed to be lower than elsewhere. Estimates from bird counts give the breeding contact rate (*b*_5_) to be a quarter of the contact rate for the rest of the year (*b*) [22]. We examine a range of transmission rates (from *b* = 0.2×10^−4^ to *b* = 2×10^−4^) so that the basic reproduction number, *R*_0_, ranges from about 0.8 to 8 (*R*_0_ = *b N/g*). In the sensitivity analysis with an infectious period of 3 days, we adjust *b* (*b* = 0.5×10^−4^ to *b* = 5×10^−4^) to retain *R*_0_ in the 0.8 to 8 range.

To allow occasional re-introduction of the virus, a background transmission rate is added to the model. This background transmission occurs when birds contract infection by any other mechanism than the ‘direct’ transmission described above. Such mechanisms include waterborne transmission, mixing with other mallard populations and mixing with other bird or animal species. Background transmission is a crucial mechanism to enable the persistence of a virus population particularly within small communities below the critical community size where epidemics cannot be sustained by direct transmission only [23]. In our model, the parameter *h* describes the rate of background transmission, which is calculated from the probability of a single duck becoming infected by background transmission in its lifetime (1% probability of infection in a mean lifetime of 828 days gives *h*≈10^−5^).

Birds recover from infection independently from regaining the ability to migrate and move from class *I*_1_ to *R*_1_, or from *I*_2_ to *R*_2_, at a rate *g*. An important aim of our model is to have an accurate description of where and when infections take place. This requires the model to accurately describe the mean time between successive generations of infected birds, i.e. the generation time or generation interval [24], [25]. In the current model formulation, with no latent period and an exponentially distributed infectious period, the mean generation time is equal to the mean infectious period [24]. In our analysis we considered two extremes of the mean generation time, our baseline choice reflecting a mild strain with longer period of virus excretion (*g* = 1/8 [26], [27]). Our second choice reflects a more severe strain with short generation time of three days (*g* = 1/3), based on experimental results and field observations [14], [28], [29].

In general the average infectious period will be shorter than the average migration delay as birds which are no longer infected may require additional time to regain the full strength required to undergo migration. The ability to migrate is regained at rate *n*, such that the average migration delay is 1/*n*. Because of the independence (in the model) between recovery from infection and regaining the ability to migrate, there is a very small probability for infectious birds to migrate. This allows the infection to spread between patches, and thus replaces more realistic mechanisms such as migration during the incubation period of the virus. We investigate a range of migration delays (from zero to 100 days). Table 1 gives an overview of all the parameters used in the model.

**Table 1:** The parameters of the model.

Symbol	Definition	Value/Range	Unit	Reference
*b_i_*	transmission rate in patch *i*	0.2×10^−4^ to 2×10^−4^ for *i*≠5; 0.05×10^−4^ to 0.5×10^−4^ for *i* = 5	bird^−1^ day^−1^	[22], [41]
*g*	recovery rate	1/8	day^−1^	[26], [27]
*b_i_*	birth rate in patch *i*	40 if 162<*t*<216 and *i* = 5; 0 otherwise	birds day^−1^	
*m*	natural mortality rate	0.315/365	day^−1^	[19]
*m_h,i_*	hunting mortality rate in patch *i*	0.320/365 for *i* = 1, 6, 7, 8; 0 for *i* = 2, 3, 4, 5	day^−1^	[20]
*m_i_*	migration rate in patch *i*	defined from Figure 1	day^−1^	
*n*	migration delay rate	1/1 to 1/100	day^−1^	[13]
*h*	environmental transmission rate	10^−5^	day^−1^	

These assumptions lead to an epidemic model consisting of five ordinary differential equations in each of the eight patches (*i*), so that for each *i* = 1, 2, .., 8 we obtain:
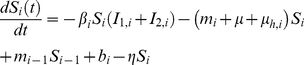
(1a)

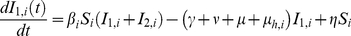
(1b)

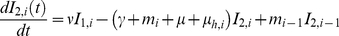
(1c)

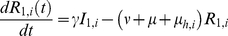
(1d)

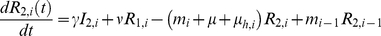
(1e)We evaluate *i* in modulus 8 such that in the wintering patch, where *i* = 1 we have, *m_i_*_−1_ = *m*_8_, *S_i_*_−1_ = *S*_8_, etc.

We wish to compare the dynamics of the above model with the behaviour in the absence of migration delay. In this case, the subclasses *I*_1_ and *R*_1_ are no longer applicable. Removing these subclasses from the model and adjusting the equations appropriately, such that infected birds directly enter class *I*_2_ when they become infected and enter class *R*_2_ when they recover, we obtain:
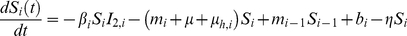
(2a)

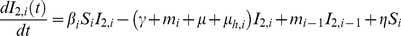
(2b)

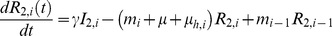
(2c)


Two sensitivity analyses were done, first with model (1) with a shorter infectious period (3 days) as described above, and second with a slightly adjusted model (1) to include loss of immunity. Loss of immunity was modelled by letting birds advance from the *R*_2_ class in patch 5 (summer) to the *S* class in patch 6 (fall_1_). Thus, under this modified scenario, each outbreak season started with a fully susceptible population.

### Simulation of model

The model is simulated in Berkeley Madonna 8.3.14 (www.berkeleymadonna.com) using the Runge-Kutta 4 method with a timestep of 0.02 days. Simulations are run over >30 years to ensure a limit cycle is reached (which occurs for all investigated parameter values).

**Figure 3:**
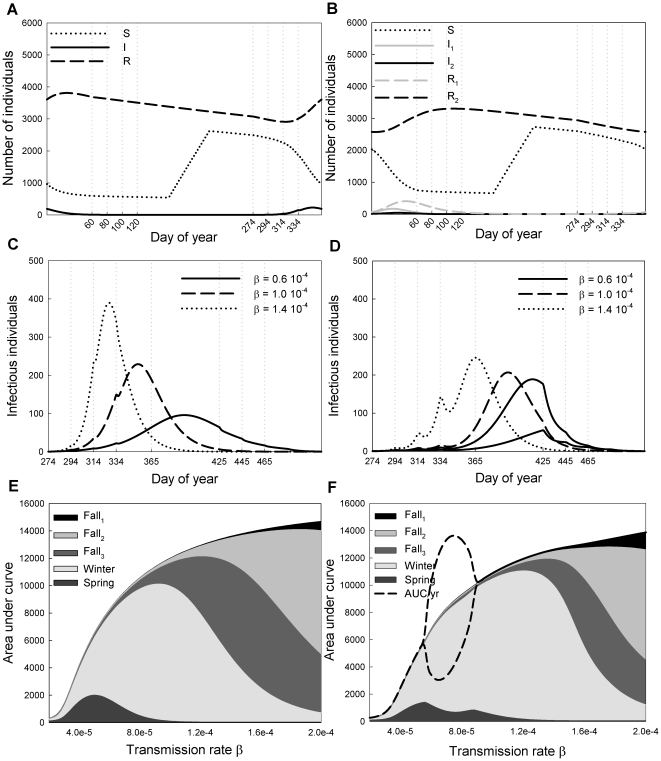
Infection dynamics with and without migration delay. The left-hand panels (A,C,E) show the dynamics of model (2), i.e. without migration delay, and the right-hand panels (B,D,F) show the dynamics of model (1) with a migration delay of 30 days. Panel (A) shows *S*(*t*) (dotted line), *I*(*t*) (solid) and *R*(*t*) (dashed) versus time for an entire year with a transmission rate of *b* = 1.0×10^−4^. The dashed vertical lines indicate the timings of migration between patches. Panel (B) shows *S*(*t*) (black dotted line), *I*_1_(*t*) (grey solid), *I*_2_(*t*) (black solid), *R*_1_(*t*) (grey dashed) and *R*_2_(*t*) (black dashed) with a transmission rate of *b* = 1.0×10^−4^. Panels (C) and (D) show *I*(*t*) = *I*_1_(*t*)+*I*_2_(*t*) for *b* = 0.6×10^−4^ (solid line), *b* = 1.0×10^−4^ (dashed) and *b* = 1.4×10^−4^ (dotted) within the three fall patches and the winter patch with dashed vertical lines to indicate the timings of migration between patches. Panels (E) and (F) show the cumulative number of daily cases of infection within a certain period, as calculated by AUC, in the three fall patches and the winter patch, versus transmission rate. The dashed curve in panel (F) indicates the total annual AUC for two subsequent years, thus showing the bi-annual pattern for a range of *b*.

As a measure for the total number of cases of infection we choose to use the measure of ‘area under the curve’ (AUC). This is numerically calculated by the area under the graph of infectious individuals versus time, such as in Figure 3C. AUC provides a better estimate than incidence of how many infected individuals are to be found at a particular patch, and therefore, of the risk of infection for other animals or populations. Incidence tells us how many individuals become infected (move from S to I) at a particular location, but neglects birds in class *I*_2_ which became infected in patch *i*−1 but have now migrated to patch *i*. The AUC measures the cumulative total number of daily cases of infection so that it scales with the infection period. If, on average, individuals are infected for eight days, they are counted once for each of these days, in total eight times.

## Results

### Model without migration delay

As a baseline, we first explore the simplified model (2) that includes birth, mortality, migration and virus transmission without any migration delay. Figure 3A shows numerical results for the typical dynamics in the absence of migration delay, using the default parameters from Table 1 and a transmission rate of *b* = 1.0×10^−4^. In most simulations with an infectious period of 8 days, the equations lead to a periodic orbit such that there is a yearly cycling which repeats indefinitely. However, for some parameters a bi-annual cycle was observed (see below). The number of susceptibles remains approximately constant during late winter and spring, until hatching begins at Day 162. During this hatching period the number of susceptibles grows linearly, reaching a peak two months before departure from the breeding site. Infection breaks out during the fall migration period, but most of the outbreak occurs during the winter, peaking around half way through. After the outbreak of infection the number of susceptibles remains approximately constant until birth begins in the subsequent year. We notice a decrease in transmission at the time of patch switch due to a reduction in the direct transmission contact rate caused by decreased numbers of birds together in the same place as a result of migration.

Both Figures 3C and 3E examine the effect of changing transmission rate upon the model without migration delay. Figure 3C shows how the number of infectious individuals varies in the fall and winter for three different values of the transmission rate. We see that as the transmission rate decreases from 1.4×10^−4^ to 0.6×10^−4^ the peak level of infection moves from fall_3_ to late winter. For all investigated parameter values we find no outbreak peak in either the spring or summer, however for *b* = 0.6×10^−4^ we see that some infection is present in the spring patches. As in Figure 3A, we notice a reduction in transmission during patch switches.

Figure 3E shows the effect of changing transmission rate on the cumulative number of daily cases of infection in each of the fall and winter patches and the spring patches combined, as calculated using the area under the curve (AUC). We see the trend alluded to in Figure 3C: as the transmission rate rises, the infection appears earlier in the year. We also see that as the transmission rate rises there are a higher total number of cases of infection per year (calculated from the sum of cases in all patches). If *b* is very low, around 0.25×10^−4^, the basic reproduction number, *R*_0_, is close to 1 and an outbreak of infection barely forms, with a maximum of 3 individuals infected at any time.

### The effect of migration delay

Figure 3B shows numerical results for the dynamics of model (1) with a migration delay of 30 days and a transmission rate of 1.0×10^−4^. Comparing Figures 3A and 3B, we see that the yearly outbreak is markedly delayed, but still occurs within the winter period. At any particular time, the majority of infectious individuals are in class *I*_1_, rather than in class *I*_2_, and are unable to migrate.

**Figure 4:**
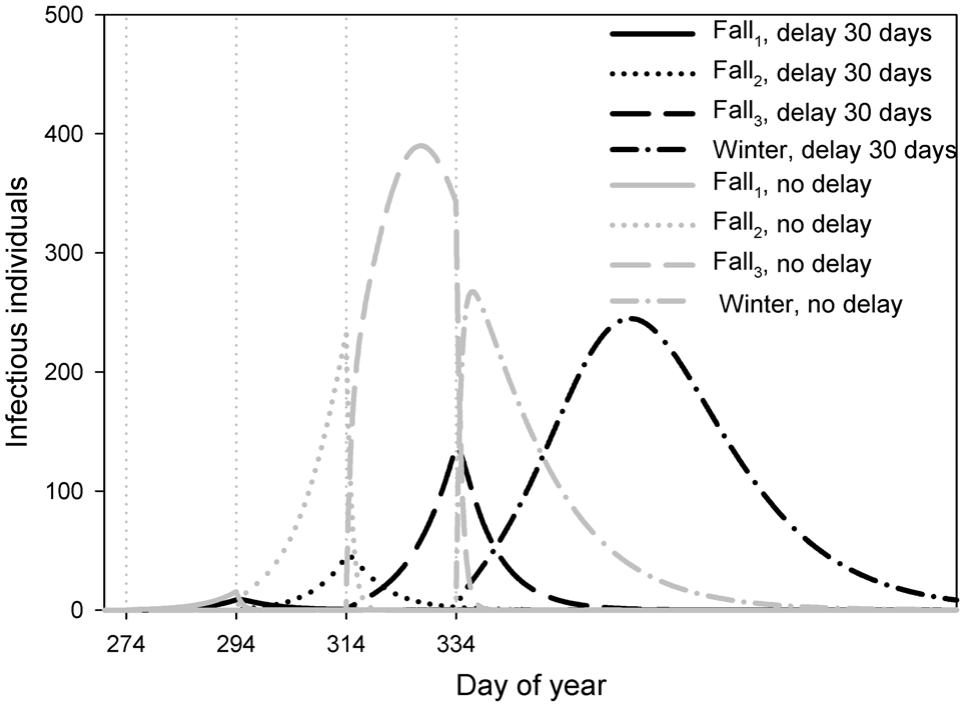
The dynamics of infection during the fall migration period. In each patch, Fall_1_ (solid), Fall_2_ (dotted) and Fall_3_ (dashed), the total number of infected individuals *I*(*t*) is plotted versus time for model (1) with a migration delay of 30 days (black lines) and model (2) with no migration delay (grey lines), for a transmission rate of *b* = 1.4×10^−4^. The dotted vertical lines indicate the timings of migration between patches.

Figures 3D and 3F examine the effect of changes in transmission rate upon the model dynamics whilst maintaining a migration delay of 30 days. Comparing Figure 3D to Figure 3C we see that infection occurs later in the year when there is migration delay. With a lower transmission rate (0.6×10^−4^), a bi-annual cycle appears, with much transmission taking place during spring migration in the years of very slow dynamics. If the transmission rate is high (1.4×10^−4^), it is clearly visible how the migration slows down transmission by decreases in prevalence just after each patch switch. In Figure 4 we see that also without migration delay there are small interruptions of virus transmission at the times of migration, but that with migration delay, in every patch a new outbreak has to develop, and only in winter, when the birds stay a few months in one patch, the outbreak can fully develop.

The shape of Figure 3F, showing the mean yearly AUC in the different patches in relation to the transmission rate, appears to be broadly similar to that of Figure 3E. Thus, in general, changes in migration delay lead to more minor variations in the dynamics of infection than those produced by changes in the transmission rate. The total number of cases of infection per year decreases due to migration delay, as a consequence of the epidemic proceeding more slowly. Furthermore we observe that the distribution of infection over the patches changes and that bi-annual cycles can appear for low values of *b*. For intermediate values of *b*, infections in winter and even spring share a higher percentage of total cases of infection (the maximal winter and spring AUCs are higher and more to the right in Figure 3F than in Figure 3E) as migration delay allows for increased infection later in the season by delaying the infection of susceptible birds. For large values of the transmission rate the migration delay seems to result in an increase in the numbers of infected birds in the earlier patches, due to infected birds remaining at these sites for longer.

### The joint action of migration delays and transmission rate

**Figure 5:**
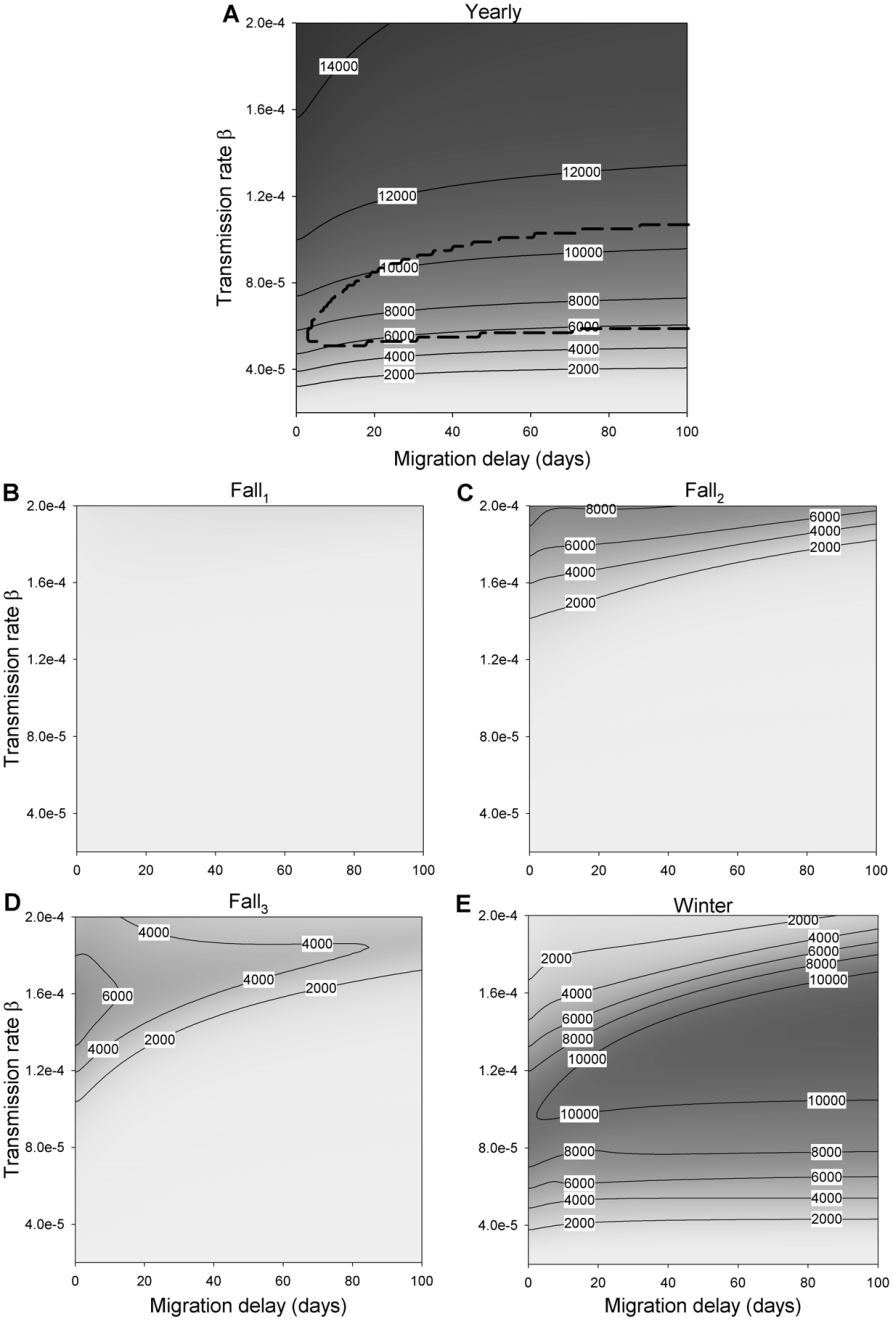
The cumulative number of daily cases of infection. The cumulative number of daily cases of infection within a certain period, both yearly (A), and in each of the four patches where infection is found, Fall_1_ (B), Fall_2_ (C), Fall_3_ (D) and Winter (E), as calculated by AUC, plotted as a function of both transmission rate and migration delay. Default parameter values, as defined in Table 1, remain constant. The area in panel (A), demarcated by a dashed curve, indicates parameter values for which bi-annual dynamics were observed.

Figure 5A shows the cumulative number of daily cases of infection (i.e. AUC) over an entire year for the full range of transmission rates and migration delays as defined in Table 1. In Figure 5A, an area is demarcated in which bi-annual dynamical patterns were observed, alternating between years with high and low AUC (the difference was never more than 1000). Dynamical patterns stretching over more than two years were not observed. The grey level shows the total mean yearly level of infection. It appears that in general, as we observed in Figure 3, the sensitivity to the migration delay (longer delay means less cases) is smaller than the sensitivity to the transmission rate (higher rate means more cases). However, the effect of delays are not negligible, as a migration delay of 30 days can lead to 20–25% fewer cases, especially with low transmission rates (0.6×10^−4^).

Figures 5B to F show how the cases are distributed over the three fall patches and the winter patch. It is clear that an increase in transmission rate brings infection earlier in the year. If the transmission rate is high and many cases occur in the fall patches, a migration delay can significantly affect the distribution over the patches. With lower transmission rates, the main effect of a transmission delay can be to create a bi-annual cycle rather than a yearly cycle. It should be noted that the Figure indicates where the infected birds will be, not when, because the increase in fall_1_ is mainly due to infected birds staying there longer.

### Sensitivity analysis

We repeated the analysis with an infectious period of 3 days instead of 8 days (Figures S1 and S2). It appears that with a shorter infectious period, outbreaks tend to be earlier in the year, even already in fall_1_, with high transmission rate (*b* = 3.5×10^−4^). The main effects of transmission rates and migration delays were not different from the baseline model, but if there are many cases in fall_1_, a migration delay can cause more cases to occur in this patch. The reason is that a migration delay reduces the total yearly incidence, resulting in more susceptible birds after summer and a faster increase in prevalence in patch fall_1_. As soon as migration to fall_2_ starts, the prevalence quickly decreases, overal resulting in fewer cases, annually. Bi-annual dynamics are still observed, though in a smaller parameter range and with less pronounced differences between the alternating years.

A second sensitivity analysis was done assuming all birds losing their immunity in patch fall_1_, just after the breeding season (Figures S3 and S4). This scenario results in much faster dynamics, with more cases in fall, and no bi-annual cycles. However, the qualitative effects of transmission rate and migration delay remain unchanged.

## Discussion

In this paper we have used a mathematical model to explore the epidemiological dynamics of avian influenza virus in a migrating mallard population with a specific view to understanding the role of delays in migration upon the spread of a virus strain of differing transmissibility. We have found that the delayed migration of individuals influences both the timing and total size of outbreaks of avian influenza virus.

Our modelling predicts that the delayed migration of infected individuals leads to a lower total number of cases of infection each year than in the absence of migration delay. This occurs as infected birds become isolated from the main population of susceptible individuals as a result of delay, leading to a reduced rate of infection at staging sites, and the epidemic proceeding more slowly. This isolation effect in turn leads to changes in the timing of outbreaks, the extent of which depends on the situation without migration delays. This situation is determined by an interplay of three model ingredients: the transmission rate, the generation time and the loss of immunity. In general, a lower transmission rate, longer generation time, and slower loss of immunity leads to more prolonged peaks with outbreaks predominantly occurring in the late fall or winter patches. Then, a migration delay further slows down dynamics, resulting in cases later in the year. When the transmission rate of a strain is high, the generation time is short, and/or immunity is lost quickly, we observe that a migration delay can slightly increase the number of cases in the first staging site (fall_1_).

The season in which most cases occur is of particular interest, as poultry densities are not equal along the migration routes [6]. Our model results show that migration delays potentially lead to a higher prevalence of infection in winter, even if those strains have a higher transmissibility. As the highest prevalences are currently seen during fall migration, migration delays could increase rates of risk contacts during winter. This could lead to a broader spread of infection, particularly if birds from other populations come into contact with infected mallards, as they may do at the wintering grounds. With very high transmission rates and a short generation time, however, the opposite may occur: because migration delays reduce the outbreak size, the increased number of susceptibles in early fall can result in a faster rate of spread and an earlier peak (Figure S1C,D).

Many current transmission risk models use solely empirical information on bird movements to assess the potential for long distance movement and, hence, transmission along a flyway [30]–[34]. In contrast, our model demonstrates that a consideration of infection-induced delays, and changes in the location of susceptibles relative to the location of the infected individuals, is critical to understanding the dynamics of infection along the entire flyway. From our results we have learnt that migration delays play a role in particular if transmissibility is limited (low *R*_0_ or a more immune population). Low transmissibility stretches the yearly outbreak over a longer period, and therefore over multiple patches, increasing the effect of migration delays. In some instances, a bi-annual pattern is observed. This is more likely if dynamics are slow, i.e. with low *b* and long migration delay. The sensitivity analyses confirm this pattern, as bi-annual cycles are more prominent if the infectious period is shorter, and disappear if immunity is not lifelong anymore. The bi-annual dynamics did not affect the mean number of cases per year, and therefore we have not further explored these dynamics. Finally, we have not taken into account additional mortality due to birds staying behind and suffering from adverse (weather) conditions. Because the role of delayed birds in spreading the virus is limited, we do not expect this assumption to affect our conclusions.

Our modelling considers the spread of virus within a single population of mallards, as the experimentally observed short shedding time of influenza virus implies that the spatial dynamics of avian influenza is mainly characterized by ‘travelling within bird flocks’ [14]. However at staging sites it is hard to imagine that there is no interaction between multiple populations and species, and there are indications that virus can survive in surface water for extended periods [35], possibly resulting in re-introductions of the infection into the population. We broadly incorporated these effects in our model via background transmission, which appears an essential model ingredient to prevent extinction of the infection [23], [36]. In a more complex model we could directly model the environment in the different patches, or the interaction between species. In particular, the occurrence of outbreaks of infection is observed to increase with colder temperatures, particularly in relation to the congregation of waterbirds along the 0°C isotherm in winter [37]. Explicit consideration of higher environmental transmission during the wintering period within a refined model could lead to predictions of outbreaks of infection in spring, as has been detected in some studies [5], and which we have not observed from our current model. Indeed it is possible that we observe no such spring outbreaks as a result of our model being deterministic such that we only predict what happens in an average population. Stochastic effects may result in different populations encountering a virus at different in times, such that the outbreaks in those populations will be later.

An important assumption is the independence between recovery from infection and regaining the ability to migrate. Although possibly biologically unrealistic, assuming a formal link between recovery status and migratory ability would have required quantitative information which is as yet unavailable. The major consequence of our current assumption is that a small number of birds (*I*_2_) is able to migrate, thus acting as seeders of a new outbreak in the next patch. Although in reality this role may not be played by birds recovered from infection, the possibility that a small number of birds bring the infection to the next patch is not entirely unrealistic, e.g. due to birds migrating during the incubation period of the virus.

Due to the uncertainty and possibly complex patterns surrounding cross-immunity in ducks our current analysis is restricted to a single immunological subtype that confers lifelong immunity. For our baseline parameter values, the seasonal dynamics produced by our host-pathogen model is a bit slow compared to experimental observations with AI virus prevalence being low during summer, peaking just after the breeding season [2] at approximately 10–20% infected birds, and then dropping during December [14]. However, there are many realistic parameter combinations that do result in the majority of cases occurring during fall: with a shorter generation time or higher transmission rate than in our baseline parameter set. In addition, in a sensitivity analysis we have considered the possibility that each fall all birds are again susceptible, due to loss of immunity or new strains circulating. This alternative assumption did not affect the conclusions of our analysis on the effects of transmission rate and migration delay. The consequence of a more gradual loss of immunity and partial cross-immunity to a new strain is more difficult to predict, and was not the aim of our study. A slow increase in susceptible birds into the population, as would be the result of waning immunity, could lead to the possibility of multiple outbreaks of infection per year, the regularity of which would depend on the relationship between the rates of waning immunity and migration delay, with a reduction in the period of immunity likely leading to more regular outbreaks.

Our model has the advantage of being general in its formulation and could be reparameterised for certain other species of migratory birds that are potential long-distance vectors of avian influenza virus such as northern pintail [38], Bewick's swans [13] or common teal [30], and for other diseases found in migratory birds [39]. We assume no age structure in our model so that both adults and juveniles behave in the same manner. Costa et al. [40] suggests that there may be an increase in the shedding rates and probability of infection between adults and immunologically naive juveniles. As our model predicts that virus prevalence peaks just after the breeding season, when a high percentage of the population consists of juveniles, we would perhaps observe more rapid spread of virus in an age structured model. The results of our model are based upon the assumptions that all birds stop at exactly three staging sites. We expect to observe the same trends in infection dynamics for any biologically realistic number of staging sites, for example, as obtained from satellite tracking studies [18], [32]. However any model which does not include staging sites, whereby birds transfer directly back and forth between breeding and wintering grounds, would fail to reproduce the predictions that we have presented here. The mean generation time in our model was assumed to be 8 days, in agreement with infectious periods in low-pathogenic AI strains [26], [27]. The general trends in our results hold for infectious periods of 3 days as observed in experimentally infected ducks [29] and more virulent strains [14], [26], [28] (Figures S1 and S2).

Experimental examination of the effect of both highly and low pathogenic avian influenza upon free-living birds is sparse. The investigations of Latorre-Margalef et al. [14] and Van Gils et al. [13] on the effect of infection upon migration behaviour are limited by their observational nature, making it impossible to separate cause from effect, and because of the dynamics of infection, particularly in the mallard population. As our model shows, a large proportion of the mallards may become infected during fall migration, meaning that although Latorre-Margelef et al. [14] compared infected with uninfected birds (at the time of capture), both groups are likely to have experienced infection that fall, potentially clouding any effect of infection on migration timing. Ideally, to test our assumptions and results, one would have a measure of recent infection, e.g. serological status, which could be compared between birds at a staging site arriving at different times during the migration season. Birds arriving late should more often show signs of recent infection than birds arriving early, according to our model.

In situations where empirical examination is hindered by the process under investigation, mathematical modelling provides a way to further investigate mechanisms and consequences of infection when there is a shortage of high quality data. Our theoretical study shows that hampered migration has the ability to alter both the timing and level of an avian influenza outbreak in wild bird populations. Further understanding of the effect of delayed migrations in wild bird populations can be achieved by additional data collection and modelling work, and as such remains a topic of interest in both theoretical and experimental epidemiology.

## Supporting Information

Figure S1 **Infection dynamics with and without migration delay, with a mean infectious period of 3 days.** The left-hand panels (A,C,E) show the dynamics of model (2), i.e. without migration delay, and the right-hand panels (B,D,F) show the dynamics of model (1) with a migration delay of 30 days. Panel (A) shows *S*(*t*) (dotted line), *I*(*t*) (solid) and *R*(*t*) (dashed) versus time for an entire year with a transmission rate of *b* = 2.5×10^−4^. The dashed vertical lines indicate the timings of migration between patches. Panel (B) shows *S*(*t*) (black dotted line), *I*_1_(*t*) (grey solid), *I*_2_(*t*) (black solid), *R*_1_(*t*) (grey dashed) and *R*_2_(*t*) (black dashed) with a transmission rate of *b* = 2.5×10^−4^. Panels (C) and (D) show *I*(*t*) = *I*_1_(*t*)+*I*_2_(*t*) for *b* = 1.5×10^−4^ (solid line), *b* = 2.5×10^−4^ (dashed) and *b* = 3.5×10^−4^ (dotted) within the three fall patches and the winter patch with dashed vertical lines to indicate the timings of migration between patches. Panels (E) and (F) show the cumulative number of daily cases of infection within a certain period, as calculated by AUC, in the three fall patches and the winter patch, versus transmission rate. The dashed curve in panel (F) indicates the total annual AUC for two subsequent years, thus showing the bi-annual pattern for a range of *b*.
(TIF)

Figure S2 **The cumulative number of daily cases of infection, with a mean infectious period of 3 days.** The cumulative number of daily cases of infection within a certain period, both yearly (A), and in each of the four patches where infection is found, Fall_1_ (B), Fall_2_ (C), Fall_3_ (D) and Winter (E), as calculated by AUC, plotted as a function of both transmission rate and migration delay. Default parameter values, as defined in Table 1, remain constant. The area in panel (A), demarcated by a dashed curve, indicates parameter values for which bi-annual dynamics were observed.
(TIF)

Figure S3 **Infection dynamics with and without migration delay, with loss of immunity at the onset of fall.** The left-hand panels (A,C,E) show the dynamics of model (2), i.e. without migration delay, and the right-hand panels (B,D,F) show the dynamics of model (1) with a migration delay of 30 days. Panel (A) shows *S*(*t*) (dotted line), *I*(*t*) (solid) and *R*(*t*) (dashed) versus time for an entire year with a transmission rate of *b* = 1.0×10^−4^. The dashed vertical lines indicate the timings of migration between patches. Panel (B) shows *S*(*t*) (black dotted line), *I*_1_(*t*) (grey solid), *I*_2_(*t*) (black solid), *R*_1_(*t*) (grey dashed) and *R*_2_(*t*) (black dashed) with a transmission rate of *b* = 1.0×10^−4^. Panels (C) and (D) show *I*(*t*) = *I*_1_(*t*)+*I*_2_(*t*) for *b* = 0.6×10^−4^ (solid line), *b* = 1.0×10^−4^ (dashed) and *b* = 1.4×10^−4^ (dotted) within the three fall patches and the winter patch with dashed vertical lines to indicate the timings of migration between patches. Panels (E) and (F) show the cumulative number of daily cases of infection within a certain period, as calculated by AUC, in the three fall patches and the winter patch, versus transmission rate.
(TIF)

Figure S4 **The cumulative number of daily cases of infection, with loss of immunity at the onset of fall.** The cumulative number of daily cases of infection within a certain period, both yearly (A), and in each of the four patches where infection is found, Fall_1_ (B), Fall_2_ (C), Fall_3_ (D) and Winter (E), as calculated by AUC, plotted as a function of both transmission rate and migration delay. Default parameter values, as defined in Table 1, remain constant.
(TIF)
