# Supplementary figures and images for: Effects of Infection-Induced Migration Delays on the Epidemiology of Avian Influenza in Wild Mallard Populations
Source: PLoS One. 2011 Oct 18;6(10):e26118. doi: 10.1371/journal.pone.0026118 (PMC3196538; doi:10.1371/journal.pone.0026118)

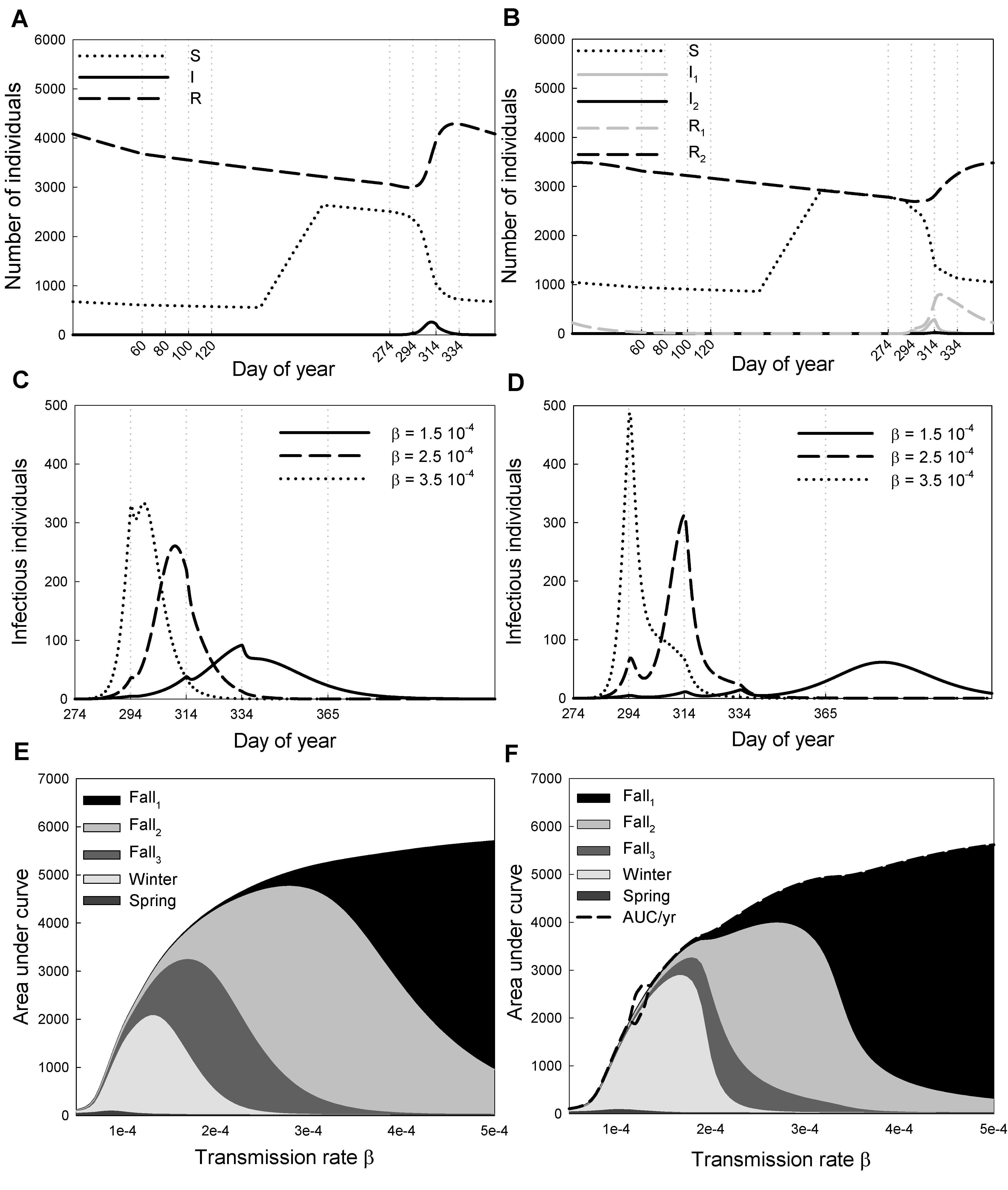

Supplement: Figure S1 — Infection dynamics with and without migration delay, with a mean infectious period of 3 days. The left-hand panels (A,C,E) show the dynamics of model (2), i.e. without migration delay, and the right-hand panels (B,D,F) show the dynamics of model (1) with a migration delay of 30 days. Panel (A) shows S(t) (dotted line), I(t) (solid) and R(t) (dashed) versus time for an entire year with a transmission rate of b = 2.5×10^−4^. The dashed vertical lines indicate the timings of migration between patches. Panel (B) shows S(t) (black dotted line), I 1(t) (grey solid), I 2(t) (black solid), R 1(t) (grey dashed) and R 2(t) (black dashed) with a transmission rate of b = 2.5×10^−4^. Panels (C) and (D) show I(t) = I 1(t)+I 2(t) for b = 1.5×10^−4^ (solid line), b = 2.5×10^−4^ (dashed) and b = 3.5×10^−4^ (dotted) within the three fall patches and the winter patch with dashed vertical lines to indicate the timings of migration between patches. Panels (E) and (F) show the cumulative number of daily cases of infection within a certain period, as calculated by AUC, in the three fall patches and the winter patch, versus transmission rate. The dashed curve in panel (F) indicates the total annual AUC for two subsequent years, thus showing the bi-annual pattern for a range of b. (TIF) [file pone.0026118.s001.tif]

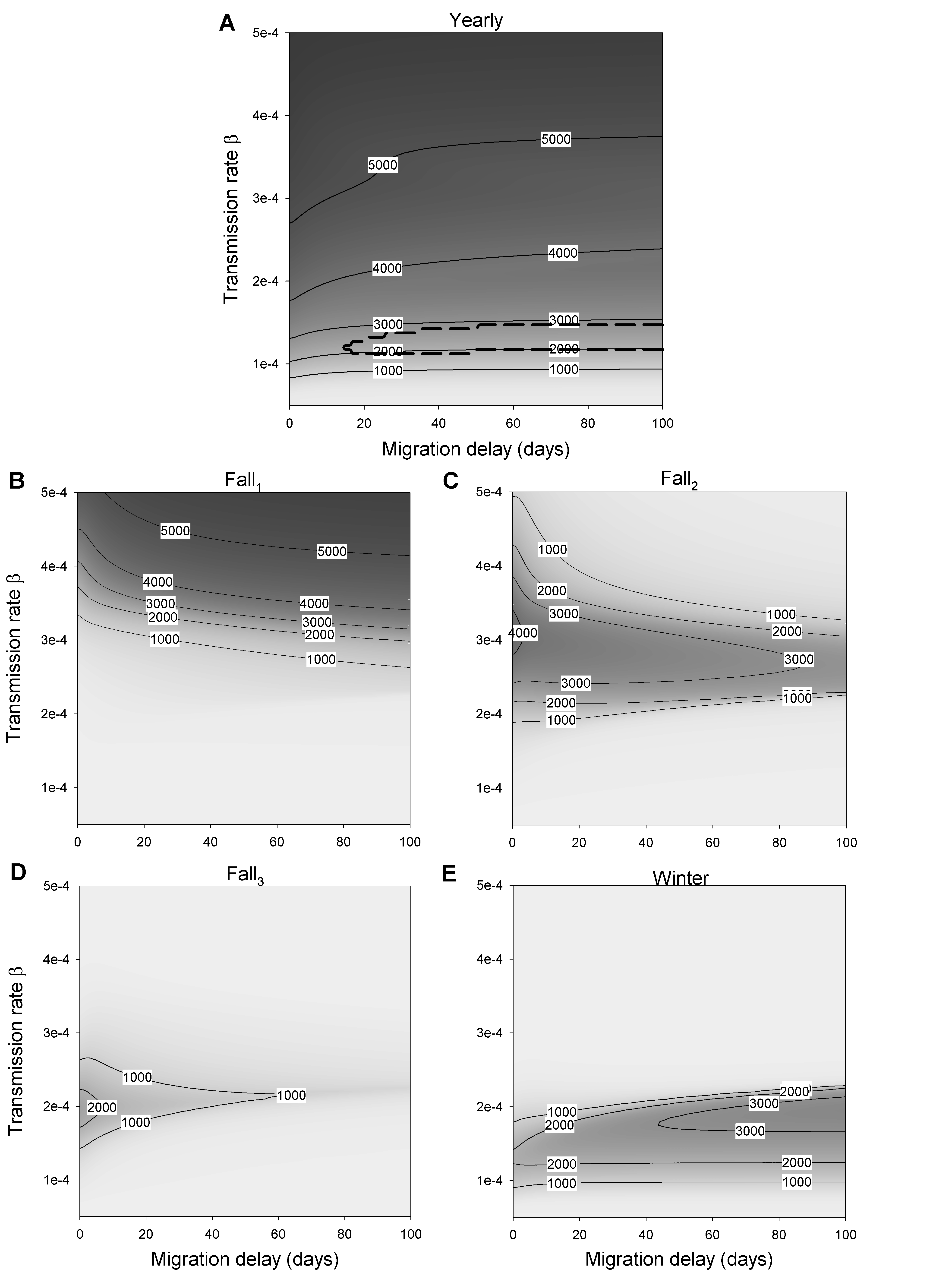

Supplement: Figure S2 — The cumulative number of daily cases of infection, with a mean infectious period of 3 days. The cumulative number of daily cases of infection within a certain period, both yearly (A), and in each of the four patches where infection is found, Fall1 (B), Fall2 (C), Fall3 (D) and Winter (E), as calculated by AUC, plotted as a function of both transmission rate and migration delay. Default parameter values, as defined in Table 1, remain constant. The area in panel (A), demarcated by a dashed curve, indicates parameter values for which bi-annual dynamics were observed. (TIF) [file pone.0026118.s002.tif]

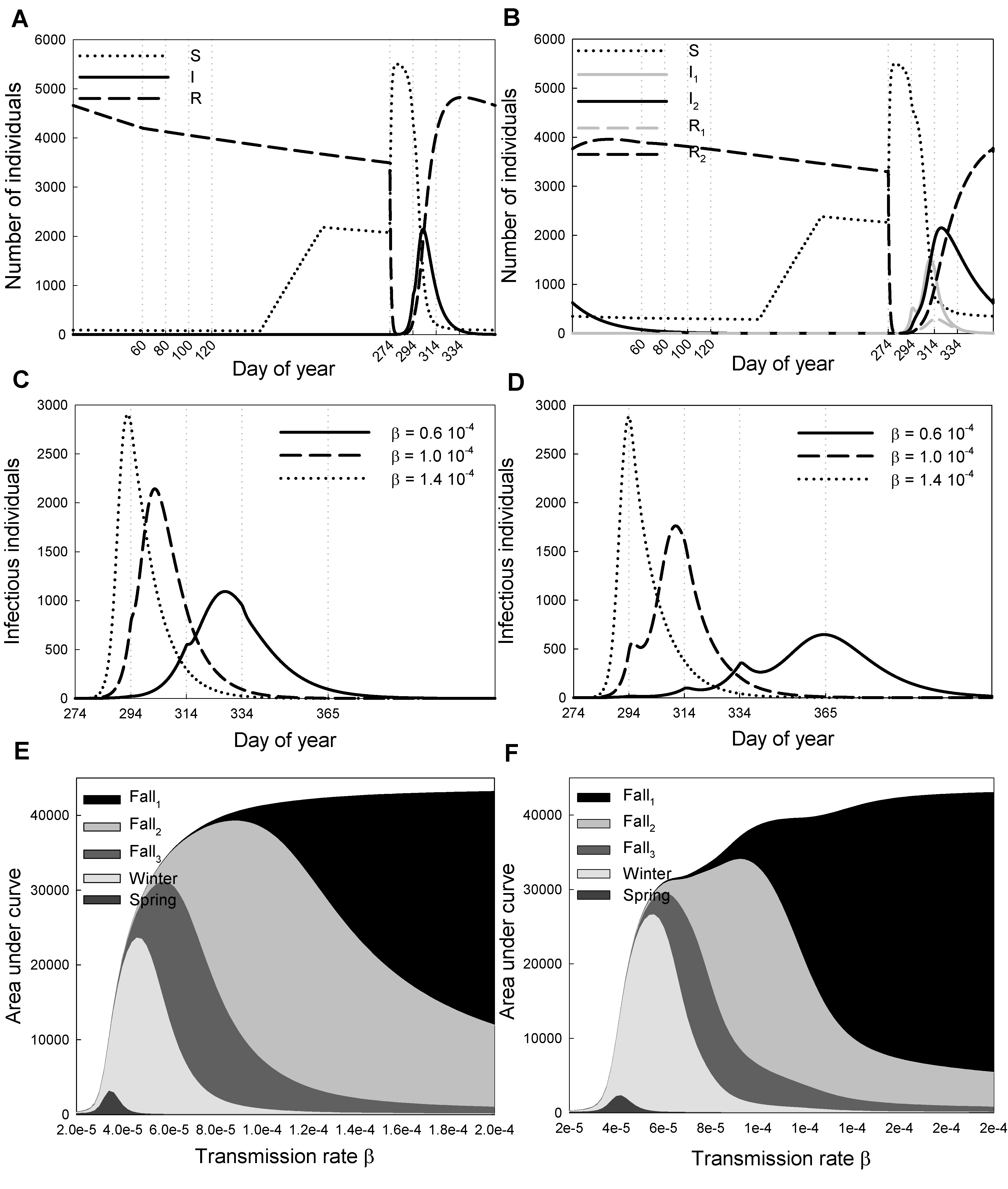

Supplement: Figure S3 — Infection dynamics with and without migration delay, with loss of immunity at the onset of fall. The left-hand panels (A,C,E) show the dynamics of model (2), i.e. without migration delay, and the right-hand panels (B,D,F) show the dynamics of model (1) with a migration delay of 30 days. Panel (A) shows S(t) (dotted line), I(t) (solid) and R(t) (dashed) versus time for an entire year with a transmission rate of b = 1.0×10^−4^. The dashed vertical lines indicate the timings of migration between patches. Panel (B) shows S(t) (black dotted line), I 1(t) (grey solid), I 2(t) (black solid), R 1(t) (grey dashed) and R 2(t) (black dashed) with a transmission rate of b = 1.0×10^−4^. Panels (C) and (D) show I(t) = I 1(t)+I 2(t) for b = 0.6×10^−4^ (solid line), b = 1.0×10^−4^ (dashed) and b = 1.4×10^−4^ (dotted) within the three fall patches and the winter patch with dashed vertical lines to indicate the timings of migration between patches. Panels (E) and (F) show the cumulative number of daily cases of infection within a certain period, as calculated by AUC, in the three fall patches and the winter patch, versus transmission rate. (TIF) [file pone.0026118.s003.tif]

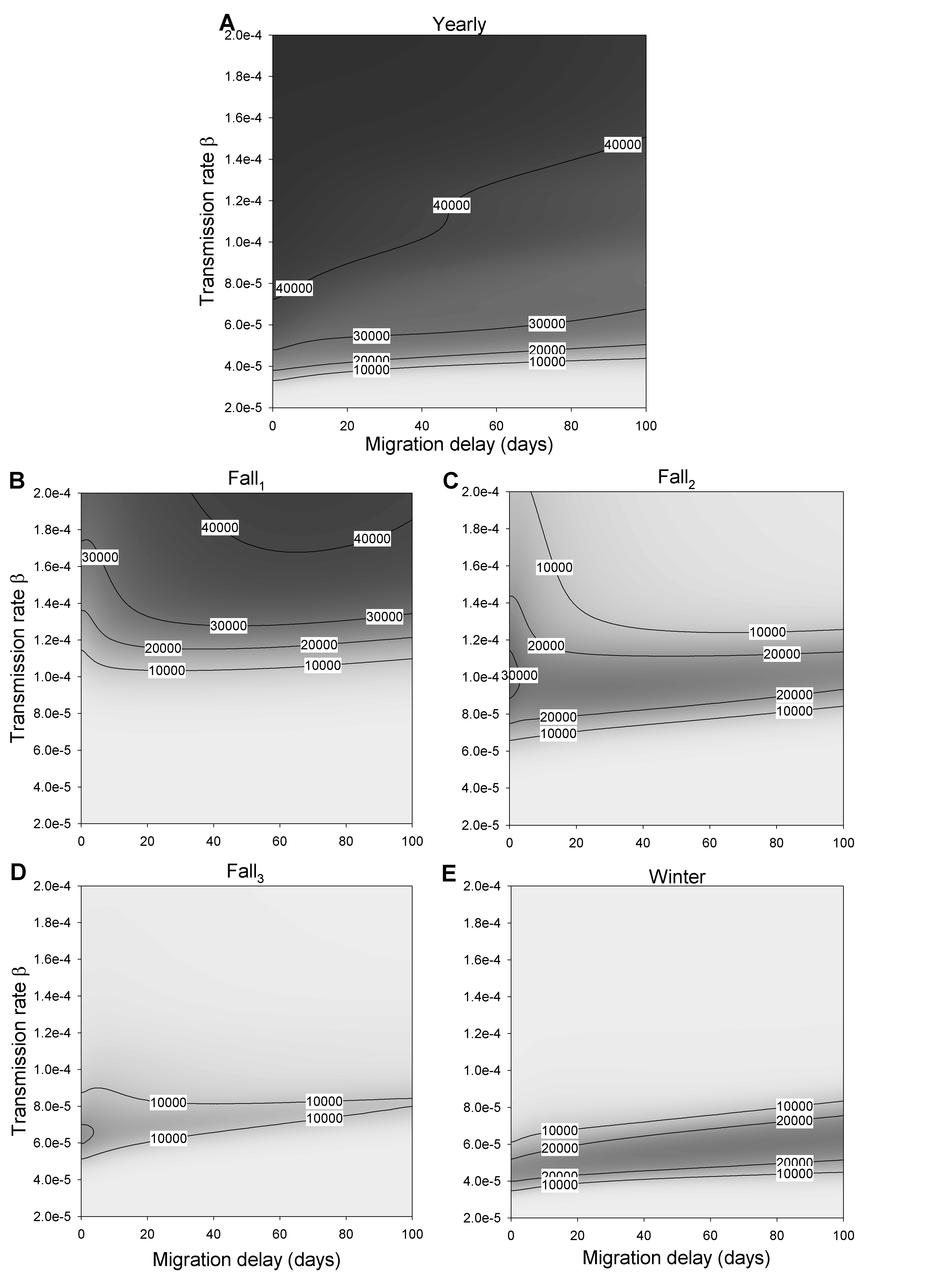

Supplement: Figure S4 — The cumulative number of daily cases of infection, with loss of immunity at the onset of fall. The cumulative number of daily cases of infection within a certain period, both yearly (A), and in each of the four patches where infection is found, Fall1 (B), Fall2 (C), Fall3 (D) and Winter (E), as calculated by AUC, plotted as a function of both transmission rate and migration delay. Default parameter values, as defined in Table 1, remain constant. (TIF) [file pone.0026118.s004.tif]
